# Representativeness of the participants in the smoking Cessation in Pregnancy Incentives Trial (CPIT): a cross-sectional study

**DOI:** 10.1186/s13063-016-1552-5

**Published:** 2016-08-26

**Authors:** Barnabas Bessing, Linda Bauld, Lesley Sinclair, Daniel F. Mackay, William Spence, David M. Tappin

**Affiliations:** 1 World Health Organization, Section of Expanded Programme on Immunization, Ground Floor, UNECA building, Box 3069, Addis Ababa, Ethiopia; 2 Centre for Tobacco and Alcohol Studies, School of Health Sciences, University of Stirling, Stirling, FK9 4LA UK; 3 Institute of Health and Wellbeing, Public Health, University of Glasgow, 1 Lilybank Gardens, Glasgow, G12 8RZ Scotland UK; 4 School of Medicine, University of Glasgow, 1 Lilybank Gardens, Glasgow, G12 8RZ Scotland UK; 5 Section of Child Health, School of Medicine, Glasgow University, Scottish Cot Death Trust, West Glasgow Ambulatory Hospital, Yorkhill, Glasgow, G3 8SJ UK

**Keywords:** Clinical markers, Cross-sectional studies, Data collection, Demography, Treatment effectiveness

## Abstract

**Background:**

The limited representativeness of trial samples may restrict external validity. The aim of this study was to ascertain the representativeness of the population enrolled in the Cessation in Pregnancy Incentives Trial (CPIT), a therapeutic exploratory study to examine the effectiveness of financial incentives for smoking cessation during pregnancy.

**Methods:**

CPIT participants (*n* = 492) were compared with all self-reported smokers at maternity booking who did not participate in the trial (*n* = 1982). Both groups were drawn from the National Health Service (NHS) Greater Glasgow and Clyde area over a 1-year trial enrolment period. Variables used for comparison were age, area-based deprivation index, body mass index, gestation, and carbon monoxide (CO) breath test level. Chi-square and Mann-Whitney *U* tests were used to compare groups.

**Results:**

From January to December 2012, 2474/13,945 (17.7 %) women, who booked for maternity care, self-reported as current smokers (at least one cigarette in the last week). Seven hundred and fifty-two were ineligible for trial participation because of a CO breath test level of less than 7 parts per million (ppm) used as a biochemical cut-off to corroborate self-report of current smoking. At telephone consent 301 could not be contacted, 11 had miscarried, 16 did not give consent and 3 opted out after randomisation, leaving 492 participants for analysis. There were no differences in demographic or clinical characteristics between trial participants, and self-reported smokers not enrolled in the trial in terms of CO breath test (as a measure of smoking level for those with a CO level of 7 ppm or higher), material deprivation (using an area-based measure), maternal age and maternal body mass index. Gestation at booking was statistically significantly lower for participants.

**Conclusions:**

To ensure that all trial participants were smokers, biochemical validation excluded self-reported smokers with a CO level of less than 7 ppm from taking part in the trial, which excluded 30 % of self-reported smokers who were ‘lighter’ smokers. The efficacy of financial incentives would not have been likely to decrease if ‘lighter’ smokers had been included in the trial population. Trial participants were slightly earlier in their pregnancy at maternity booking, but this difference would not clinically affect the provision of financial incentives if provided routinely. Overall, the trial population was representative of all self-reported smokers with regard to available routinely collected data. Appropriate comparison of trial and target populations, with detailed reporting of exclusion criteria would contribute to the understanding of the wider applicability of trial results.

**Trial registration:**

Current Controlled Trials ISRCTN87508788. Registered/Assigned on 1 September 2011.

## Background

The fundamental evidence for decision-making in patient care has in the past been based on clinical experience [1]. More recently, clinicians and decision-makers have come to accept that rigorous research is required in addition to experience [2] in order to tackle growing population health challenges. Current intervention protocols and clinical guidelines combine high-quality research from individual patient treatment [3] with clinical experience. Randomised controlled trials (RCTs) with reliable internal validity are considered the ‘gold standard’ [4] to inform decision-making for both individual patients and population health [5–7]. Guidelines to help clinicians make quick but accountable decisions should be guided by accurate and reliable research findings from RCTs [3] to minimise bias. However, trial findings must benefit the target group with similar therapeutic needs (external validity) [4]. Clinicians [8–10] have questioned the effectiveness and reliability of applying trial evidence to target populations outside a trial setting. This may explain delayed use of trial evidence in clinical practice [10, 11]. In the USA, a large body of research has been reviewed showing that ethnic minority groups are under-represented in trials [12]. Much of this review focussed on reasons why ethnic minority groups are more difficult to access and are less likely to agree to take part and be retained in research studies. There are many plausible reasons, but in general trials have insufficient representation from ethnic minority groups for clinicians to feel confident that the trial results should lead to change in treatment for those groups.

Trial selection criteria [6, 13, 14] and overall methodology to ensure internal validity may threaten representativeness. For instance, patients with comorbid conditions are often excluded from trials. In subsequent clinical practice, beneficial effects may not be realised perhaps due to side effects associated with a common comorbid condition that is highly represented in the target population. It is important, therefore, to compare trial participants with the target population with regard to demographic, clinical and other variables. Such comparison will help to uncover possible bias associated with nonrepresentative trial populations and allow a judgement to be made about the likely generalisability of trial results [4, 15]. Models of reasons for taking part and not taking part are important [16] to try to understand how better to run research projects that are representative and, therefore, produce results applicable to all groups in the target population. However, this present study did not attempt to understand the reasons behind lack of representativeness, but was limited to establishing representativeness or not, using limited criteria available from both the trial population and the target population from which the trial population was drawn.

The Cessation in Pregnancy Incentives Trial (CPIT) [17, 18] was a therapeutic exploratory phase II trial to examine the efficacy and acceptability of using financial incentives to help pregnant smokers to make use of routine Stop Smoking Services (SSS) and or quit smoking during pregnancy. Women were eligible if they were self-reported smokers with an exhaled carbon monoxide level of at least 7 parts per million (ppm) (to biochemically demonstrate current smoking), aged 16 years or older (for consent), less than 24 weeks pregnant (to allow intervention lasting 12–16 weeks by SSS), resident in NHS Greater Glasgow and Clyde (to allow access for research nurses to collect biochemical verification measures of smoking cessation), and able to understand and speak English (for telephone consent). This study used 492 trial participants of 2494 (20 %) women who self-reported as current smokers (at least one cigarette in the last week) at their first maternity booking contact appointment – the target population. The trial showed the intervention to be effective [18] and cost-effective [19]; however, there remains a valid question: ‘Is the intervention likely to be effective if applied to the whole target population?’

**Fig. 1:**
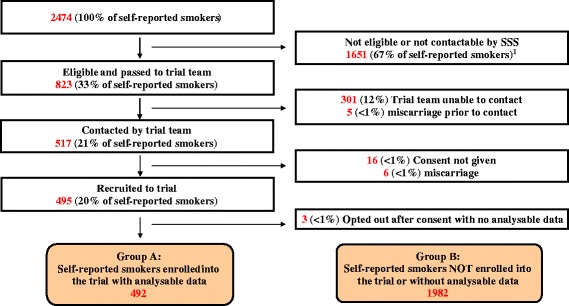
Study sampling frame and sample. Two thousand four hundred and seventy-four women between January and December 2012 replied ‘Yes’ when asked by their midwife at their maternity booking visit if they had smoked at least one cigarette in the last week. Seven hundred and fifty-two had a carbon monoxide level (CO) less than 7 ppm, or unavailable in 172 (7 %); 13 (<1 %) were under 16 years of age; 243 (10 %) had a gestation ≥24 weeks; 593 (24 %) were not contactable by the Stop Smoking Service (SSS); 23 (<1 %) were non-English speaking; the trial was not discussed by the SSS with 31 (1 %); and permission was not obtained to pass contact details for 113 (5 %). These categories are not mutually exclusive. The remaining 823 (33 % of all self-reported smokers) were eligible for the trial and were passed to the trial team. A records check showed that five had miscarried and contact was not attempted. Three hundred and one could not be contacted by the trial team. Of the 517 contacted, 6 had miscarried and 16 did not consent to the trial. Of the 495 who consented and were randomised, 3 control participants opted out and did not want any data collected in the trial to be used leaving 492 trial participants with data for analysis making up group A. All self-reported smokers who were not included in the trial analysis (2474–492) made up group B (*n* = 1982)

Comparisons were, therefore, made between self-reported pregnant smokers not included in the trial or without analysable data (group B; Fig. 1) and trial participants enrolled in CPIT with analysable data (group A; Fig. 1) [17, 18]. Together these groups make up the target population for the intervention. The findings will help policy-makers to determine the representativeness of the trial population and the likely generalisability of financial incentives for smoking cessation during pregnancy.

## Methods

The design of this representativeness study was cross-sectional, comparing the trial population with self-reported pregnant smokers not included in the trial in terms of available routinely collected demographic and clinical data. The setting was maternity booking in early pregnancy in NHS Greater Glasgow and Clyde in the West of Scotland where all pregnant women are routinely asked about their smoking status and a CO breath test is administered as an objective proxy measure of smoking status – about 97 % of pregnant women at first maternity booking in Greater Glasgow and Clyde undergo this test [20]. Client details, including self-reported smoking status and CO level are forwarded using an opt-out approach from the maternity booking appointment to the NHS SSS as recommended in national guidelines [21]. The SSS then telephones all pregnant smokers to discuss smoking and attempting to quit. During this contact, verbal permission was obtained from eligible potential trial participants to pass their contact details to the trial team. Ineligible pregnant smokers were those: with a CO breath test level less than 7 ppm or where no CO breath test level was available; where no contact was possible by the SSS; who were under 16 years of age; who were at 24 full weeks gestation or more at maternity booking (to allow SSS support to take place prior to expected delivery); who were not approached by SSS about the trial; who did not speak English; and who refused permission to pass contact details to the research team. The trial team attempted to contact pregnant smokers to discuss trial participation and a few who were contacted were no longer pregnant. The remaining patients were asked for verbal consent to take part in the trial. Those who agreed to take part were enrolled into the trial.

Group A are trial participants (*n* = 492). Group B are self-reported smokers at maternity booking who were not included in the trial when analysed (*n* = 1982).

### Variables used to compare group A with group B

Variables from the two population groups were gathered from routinely collected maternity service data: CO breath test level, height, weight, postcode, age and gestation. Gestation was recorded as estimated gestation (in weeks calculated from recall of last menstrual period) at the date of referral to the SSS and ranged from 4 to 40 weeks. Body mass index (BMI) was derived from maternal height and weight using the formula;

$$ \mathrm{W}\mathrm{t}\ \left(\mathrm{in}\ \mathrm{kilos}\right)/{\mathrm{Ht}}^2\left(\mathrm{in}\ \mathrm{metres}\right). $$

Finally, material deprivation was measured using the Scottish Index of Multiple Deprivation (SIMD) [22] with postcode as proxy. ‘SIMD ranks small areas (called datazones) from most deprived (ranked 1) to least deprived (ranked 6505) using 38 indicators from seven domains; income, employment, health, education, skills and training, housing, geographic access and crime’ [22]. In order to compare the CO levels (and, therefore, heaviness of smoking [23]) between those enrolled in the trial and those who chose or could not be enrolled in the trial, comparison between groups A and B was made after excluding those with a CO level less than 7 ppm. The aim was to show that apart from the exclusion criterion of a CO level less than 7 ppm the groups were similar in heaviness of smoking.

### Statistical analyses

Statistical analyses were performed with Stata 12.10 [24]. Descriptive statistics (percentages, medians and interquartile ranges) were derived for all variables. Univariate statistical comparison used Pearson *X*^2^ test for linear trend and Mann-Whitney *U* tests to ascertain possible group differences between group B – self-reported smokers not included in the trial and group A – the trial participants. We used multivariable logistic regression to control for the possible effects of confounding among our variables. Nonlinearities for continuous variables were tested using polynomial terms.

## Results

### Study setting and sample characteristics

Of the women who booked for maternity care, 2474/13,945 (17.7 %) were self-reported smokers, 172 (7.0 %) were excluded from all analyses because their CO level was missing or unknown, 752 (30.4 %) were excluded because their CO level was below 7 ppm, and 727 (29.4 %) did not meet the other inclusion criteria (Fig. 1). After checking routine records, 5 (<1 %) had miscarried and, therefore, contact was not attempted, and for 301 (12 %) contact was unsuccessful. Out of the 517 women contacted, consent for enrolment in the trial was not given by 16 women, 6 had miscarried, and the remaining 495 were enrolled. Three participants withdrew, refusing permission for their data to be used, leaving 492 trial participants’ data for analysis (Fig. 1) as group A. Those not included in the trial, 1982 (2474 − 492), made up the comparison group B.

### Comparison of groups

The median CO breath test result for both self-reported smokers at maternity booking not included in the trial (excluding those with a CO level less than 7 ppm) and the trial participants was 12 ppm (Table 1). A statistically significant difference (*P* < 0.001) was seen for gestation at booking with those in the trial booking at slightly earlier gestation (median 12.9 weeks compared with 13.3 weeks for those not included in the trial).

**Table 1:** Summary of baseline characteristics of nontrial (group B) and trial (group A) groups taken from all self-reported smokers at maternity booking from January to December 2012

Characteristics	Nontrial group B (*n* = 1982)	Trial group A (*n* = 492)	*P* value
CO level (ppm) for those with CO ≥7 ppm, median (interquartile range)	12 (10–16)	12 (10–17)	0.98
Gestation (weeks), median (interquartile range)	13.3 (11.7–15.3)	12.9 (11.3–14.3)	<0.001^a^
Deprivation quintiles, *n* (%)			
Most deprived			
1st	1331 (67.4)	322 (65.5)	0.58^b^
2nd	319 (16.2)	83 (16.9)	
3rd	168 (8.5)	51 (10.4)	
4th	100 (5.1)	21 (4.3)	
5th	56 (2.8)	15 (3.1)	
Least deprived			
Missing	8	0	
Age under 20	272 (13.7)	59 (12.0)	0.09^c^
20–24	599 (30.3)	126 (25.6)	
25–29	491 (24.8)	136 (27.6)	
30–34	359 (18.1)	113 (23.0)	
35+	258 (13.0)	58 (11.8)	
Missing	3	0	
BMI categories, *n* (%)			
Underweight	90 (4.6)	19 (4.0)	0.15^d^
Normal weight	921 (47.5)	215 (44.7)	
Overweight	532 (27.5)	139 (28.9)	
Obese	395 (20.4)	108 (22.5)	
Missing	44	11	

^a^Mann-Whitney *U* test

^b^Chi-square test of trend

^c^Chi-square test of trend

^d^Chi-square test of trend

*BMI* body mass index, *CO* carbon monoxide

Table 2 shows the odds ratios for a multivariable logistic regression of trial status on gestation, age category, deprivation category and BMI category. All variables with the exception of estimated gestation are not statistically significant. The effect of estimated gestation is that of increasing the odds into the trial. However, the square term shows that at higher gestational ages the odds of being in the trial declines significantly, *P* = 0.007.

**Table 2:** Odds ratios (ORs) for a multivariable logistic regression of trial status on gestation, age category, deprivation category and body mass index (BMI) category

Characteristics	OR (95 % CI)	*P* value
Gestation (weeks)	1.12	0.122
Gestation (squared)	0.99	0.007
Deprivation quintiles, *n* (%)		
Most deprived		
1st	1.0	
2nd	1.13 (0.86, 1.49)	0.383
3rd	1.25 (0.89, 1.77)	0.201
4th	0.81 (0.50, 1.33)	0.416
5th	1.08 (0.59, 1.96)	0.806
Least deprived		
Age		
under 20	1.0	
20–24	0.91 (0.64, 1.29)	0.590
25–29	1.20 (0.85, 1.71)	0.301
30–34	1.34 (0.93, 1.93)	0.117
35+	1.10 (0.72, 1.67)	0.651
BMI categories, *n* (%)		
Underweight	1.0	
Normal weight	1.19 (0.70, 2.03)	0.526
Overweight	1.38 (0.80, 2.39)	0.251
Obese	1.35 (0.77, 2.37)	0.288

## Discussion

### Comparing trial participants and self-reported smokers not included in the trial

Overall, there was no evidence from available routinely collected demographic or clinical characteristics that trial participants differed in a way that would make a difference to implementation or effectiveness of the incentives intervention. This suggests that the trial sample was representative of the whole target population of self-reported smokers identified at maternity booking in NHS Greater Glasgow and Clyde (Table 1). This is similar to randomised controlled trial studies in cannabis dependence [25] and also in smoking and chewing tobacco cessation trials [26, 27] none of which were among pregnant women. However, other studies [28, 29] found their trial populations to be nonrepresentative in terms of demographic and clinical characteristics. Evidence from systematic reviews [8, 30] indicates that pharmaceutical trials often exclude the majority of patients based on age, comorbidity, multiple drug use and other unexplained reasons, resulting in nonrepresentative samples. Our results indicate that the CPIT population was a representative sample of all self-reported smokers identified at first maternity booking contact visit (the target population) in NHS Greater Glasgow and Clyde Health Board area.

An exhaled CO level of less than 7 ppm excluded 30 % of self-reported smokers. The aim was to make sure that all participants were smokers rather than nonsmokers masquerading as smokers to try to receive incentive payments. A previous study in the same geographical area [31], before the incentives trial, showed that 36 % of self-reported smokers at maternity booking (with no advantage gained by calling themselves smokers) had a CO level of less than 7 ppm. It therefore seems likely that self-reported smokers who were excluded because their CO breath test gave a reading less than 7 ppm were true smokers but light smokers [23]. Light smokers are more likely to successfully quit with effective support [32]. Therefore, excluding these women is not likely to have overestimated the true effectiveness of financial incentives if offered to all self-reported smokers identified at maternity booking.

Gestation was significantly different with self-reported smokers who were not included in the trial, having a higher gestation at maternity booking of 13.3 weeks compared to trial participants’ 12.9 weeks. However, this difference is small and likely to be skewed by the few women who book late during pregnancy as women with greater than 24 weeks gestation at maternity booking were also excluded. This difference in gestation between groups A and B would not be large enough to affect implementation of financial incentives if they were rolled out across the health board area.

Achieving representativeness and external validity alongside internal validity is a difficult task especially in clinical trials. This study, using data from a novel trial for smoking cessation in pregnancy, showed that routinely collected data from maternity booking can provide large general target and trial population groups for adequate comparison, essential when assessing representativeness. Data items available for this study were by no means exhaustive, but those available show that participants were of similar age, material deprivation status, heaviness of smoking addiction and gestation to those women who did not take part from the target population. Therefore, it seems likely that an effective smoking cessation intervention demonstrated among trial participants would be generalisable to the whole of the target population of pregnant smokers identified at the first maternity booking visit.

### Limitations of the study

Consideration of other important covariates with regard to representativeness of the trial population which were not available – such as marital status, education, partner smoking status, employment, symptoms of psychological distress, and others known to influence smoking during pregnancy – may have produced different results.

Reasons why the study population might not be representative such as ‘program benefits’ and ‘barriers to participation’ [16] were not considered but are of great importance when beginning to design a public health intervention strategy and through the stages of testing the intervention – pilot, definitive trial and implementation.

### Implications for practice

To help assess applicability and to enable clinicians to replicate intervention strategies to improve health, researchers can make use of available routinely collected data in intervention studies in a relatively inexpensive way. This would allow assessment of the representativeness of the study population compared with the target population from which it was drawn and for whom the intervention is planned. Detailed reporting of reasons for exclusions and presentation of results by age, sex, socioeconomic status and other important characteristics, such as ethnic group, could help clinicians and policy-makers to take more informed decisions on whether to implement interventions.

Enrolment and retention or not in public health intervention trials should be modelled carefully at every stage of the research process from design through pilot and definitive trial stages to the end of implementation [16]. In this way, barriers to participation and retention in trials which often mirror barriers to implementation can be understood and removed if possible at an early stage. If barriers cannot be removed or the program benefits cannot be effectively enhanced to overcome barriers, then public health interventions can be abandoned or redesigned before large amounts of public money are spent on interventions that will not be effective for the target population.

## Conclusion

This study shows that the trial population who took part in the Cessation in Pregnancy Incentives Trial (CPIT) were representative of the target population of pregnant smokers identified at maternity booking in the trial catchment area in relation to important demographic and clinical variables. This finding supports the view that the effectiveness of the intervention examined in the trial would be generalisable if offered to all pregnant smokers identified in the trial area at maternity booking and perhaps more widely. To underpin guidelines relevant to the majority of the population that call for quality, efficient, effective and holistic health care, trial participant representativeness to the target population should be examined alongside internal validity in the ranking of evidence.

## Abbreviations

BMI: Body mass index
CO: Carbon monoxide
CPIT: Cessation in Pregnancy Incentives Trial
NHS: National Health Service
ppm: Parts per million
RCT: Randomised controlled trial
SSS: NHS Stop Smoking Service
